# Comparison of dosimetric characteristics of Siemens virtual and physical wedges for ONCOR linear accelerator

**DOI:** 10.4103/0971-6203.62137

**Published:** 2010

**Authors:** Ehab M. Attalla, H. S. Abo-Elenein, H. Ammar, Ismail El-Desoky

**Affiliations:** 1 National Cancer Institute, Cairo University; Egypt; 2 Children’s Cancer Hospital, Egypt

**Keywords:** Physical wedge, virtual wedge, wedge, wedge dosimetry

## Abstract

Dosimetric properties of virtual wedge (VW) and physical wedge (PW) in 6- and 10-MV photon beams from a Siemens ONCOR linear accelerator, including wedge factors, depth doses, dose profiles, peripheral doses, are compared. While there is a great difference in absolute values of wedge factors, VW factors (VWFs) and PW factors (PWFs) have a similar trend as a function of field size. PWFs have stronger depth dependence than VWF due to beam hardening in PW fields. VW dose profiles in the wedge direction, in general, match very well with those of PW, except in the toe area of large wedge angles with large field sizes. Dose profiles in the nonwedge direction show a significant reduction in PW fields due to off-axis beam softening and oblique filtration. PW fields have significantly higher peripheral doses than open and VW fields. VW fields have similar surface doses as the open fields, while PW fields have lower surface doses. Surface doses for both VW and PW increase with field size and slightly with wedge angle. For VW fields with wedge angles 45° and less, the initial gap up to 3 cm is dosimetrically acceptable when compared to dose profiles of PW. VW fields in general use less monitor units than PW fields.

## Introduction

The use of wedge filters in radiotherapy to produce dose gradients across the beam profile is widespread. Traditionally this is done using physical wedges made of metallic material shaped in such a way as to produce graduated attenuation across the radiation beam.[1]

Using wedge filters can improve the dose uniformity in the target volume. They can be used as missing tissue compensators and as a wedge pair to alter the shape of isodose curves so that two beams can be angled with a small hinge angle at the target volume without creating a hot spot. In recent years, digital linear accelerators have made it possible to create wedged dose distributions by computer-controlled collimator motion. The report of first implementation of dynamic wedge (DW) by Leavitt *et al*. was published in 1990.[2] Recently, Siemens has introduced a virtual wedge (VW) that creates wedge-like dose distribution by motion of one of the collimator jaws across the field during irradiation. The speed of the jaw motion is constant for a given VW field but the dose rate changes. VW was designed to mimic dosimetric properties of the physical wedges (PWs) as close as possible.[4] Due to different mechanisms used to generate wedged dose distribution and their positions relative to the linear accelerator target, the two wedge systems PW and VW are expected to have some different dosimetric characteristics. There are extensive studies on PW[4–13] and a number of studies on DW[2,14–19], enhanced dynamic wedge (EDW),[20–24] and VW.[3,25–30] In this paper, dosimetric properties of VW and PW, including wedge factors (WFs), depth doses, dose profiles and peripheral doses, are compared.

## Materials and Methods

All measurements were performed on a Siemens ONCOR linear accelerator with 6- and 10-MV X-ray beams. The linear accelerator head contains two jaw sets y1 and y2 to define the field size in the in-plane direction and two multi-leaf collimator (MLC) sets ×1 and ×2 to define the field size in the cross-plane. Each MLC set consists of 41 leaves of 1-cm resolution at the linear accelerator isocenter. The MLC is double focused. A three-dimensional scanning water phantom dosimetry system measuring 40 × 40 × 40 cm, PTW, Germany, was used to scan depth doses and dose profiles for open and physical wedge fields. An LA48 linear chamber array detector (PTW-LA48) was used for VW profile measurements. The LA48 linear chamber array consists of 47 ionization chambers arranged in one row with resolution of 0.8 cm.

Depth doses for VW fields were measured point by point at selected depths (0.5, 1, d _max_, 5, 10, 20 and 30 cm). For each VW angle, we set up the collimator to 20 × 20 cm^2^ and delivered 100 MU. Dose was measured at the first depth (0.5 cm) on the central axis by the PTW 30013 Farmer-type ion chamber of volume 0.6 cc. For the other depths on the central axis, the dose was measured as for the first depth; then, all point doses were renormalized to the dose at d _max_ . The 3D water phantom equipped with an LA48 linear chamber array detector (PTW-LA48) was used for VW profile measurements to field size 20 × 20 cm^2^ . For all the measurements with the scanning water phantom, the surface of the water phantom was placed at the source-to-surface distance (SSD) of 100 cm. Peripheral doses were derived from normalized dose profiles at the depth of maximum dose. Data presented for a quantitative comparison are for the positions 5 cm away from the field edges at the heel and toe sides of the wedge field. Wedge factors as a function of depth were obtained from ratios of depth doses for wedge fields to those for open fields. Wedge factors as a function of field size were measured with a PTW 30013 Farmer-type ion chamber in a water phantom at a depth of 10 cm and SSD of 90 cm for field sizes 5 × 5, 10 × 10, 15 × 15, 20 × 20 and 30 × 30 cm^2^ . Surface dose percentage for open and wedged fields was recorded from the measured depth dose curves for open and wedged fields.

The basic dosimetric principles of VW have recently been presented by van Santvoort.[3] The monitor unit (MU) in the wedge direction can be analytically described by an exponential function,

........(1), $$\mathrm{MU}\;\left(Y\right)\;=\;\mathrm{MU}\left(0\right)\;\exp\left(\mathrm{cµ\gamma}\;\mathrm{tan\alpha}\right)$$

where MU(*Y*) is the number of monitor units given while a point at position *Y* is within the field, MU(0) is the number of monitor units at the central axis (*Y*=0), α is the nominal wedge angle, *c* is the mean linear attenuation coefficient calibration factor or, simply, the calibration factor, and *μ* is the default mean linear attenuation coefficient.

## Results

**Figure 1:**
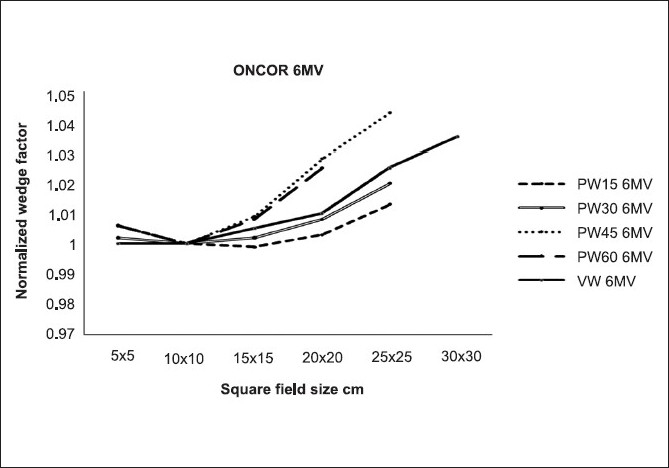
A comparison of field size dependence of normalized wedge factors

Figure 1 shows the normalized WFs for 15°, 30°, 45° and 60° VW and PW fields as a function of field sizes for 6- MV X-ray beams. Similar results have been observed for all wedge angles at 10 MV. The results showed that for PW there was a small increase in WF with field sizes (FS) for 6 and 10 MV. For VW there were no differences for 6 MV with FS, but at 10 MV there were minor differences not exceeding 1%.

Figure (2a–f) displays the percent depth dose (PDD), or the depth dependence of dose for open, VW and PW fields, with the field size of 20 × 20 cm^2^ . For the 6-MV X-ray beam, Figures 2A–2C show that, the open field has the smallest PDDs, the VW field has slightly larger PDDs and the PW field has the largest PDDs . The difference in PDDs between open, VW and PW fields decreases as the wedge angle decreases. For 10-MV photon beams, the surface doses were 37% and 47% for wedge field and open field, respectively and these were lower than that of 6-MV photon beams [Figure 2]. Surface doses for VW fields were the same as those for PW fields. Surface doses for the PW and VW fields were in general less when compared with those for open fields. At the SSD of 100 cm, the reduction was approximately 10% and 5%, observed for 30°, 45° and 60° wedge angles with the field size of 20 × 20 cm^2^ in the 6- and 10-MV beams, respectively.

**Figure 2: (a-c):**
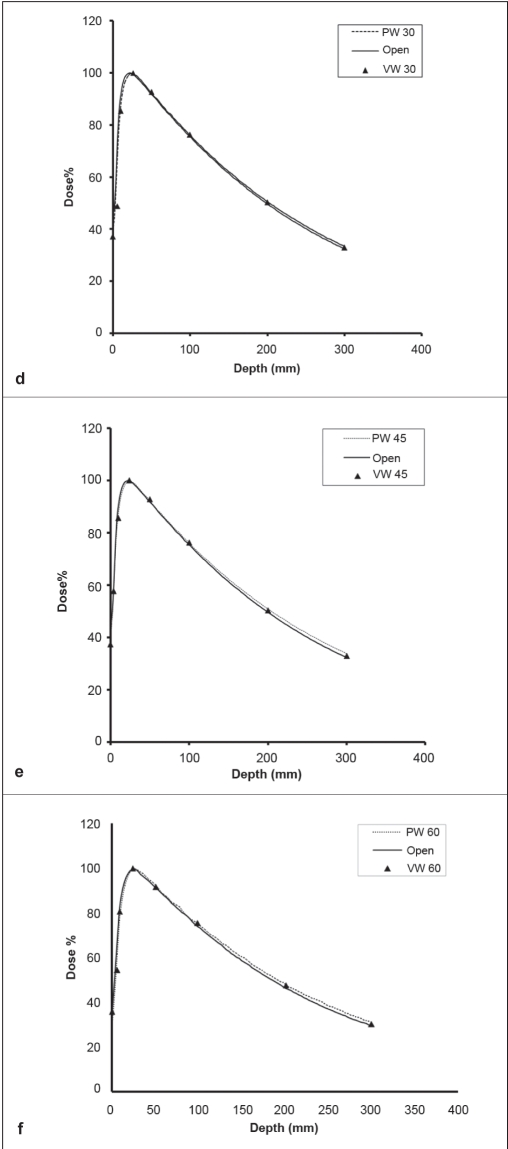
A comparison of depth doses for open, VW and PW fields with a field size of 20 × 20 cm2 for 30°, 45°, 60° wedges in 6-MV beam

**Figure 2: (d-f):**
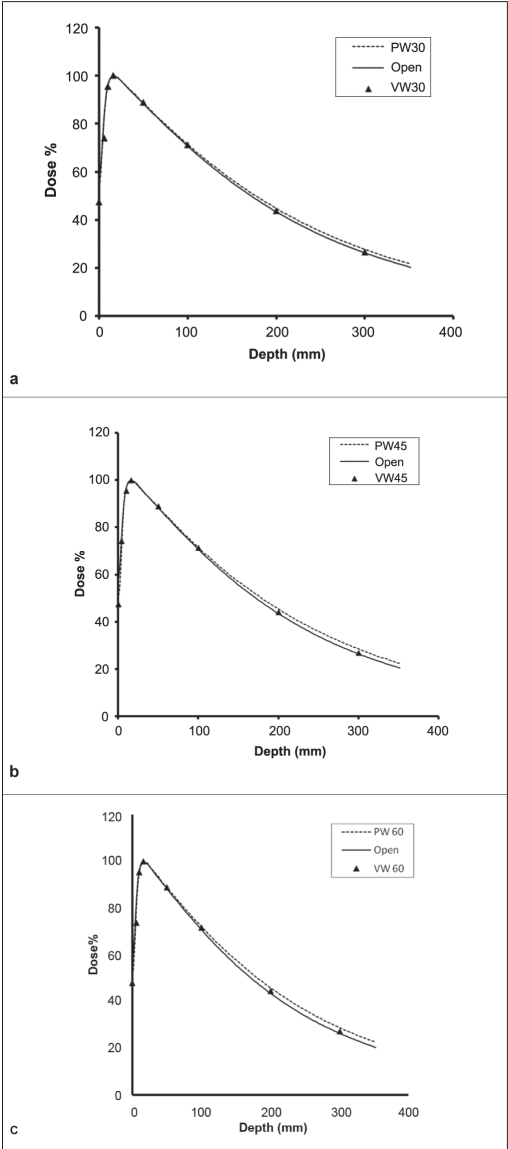
A comparison of depth doses for open, VW and PW fields with a field size of 20 × 20 cm2 for 30°, 45°, 60° wedges in 10-MV beam

**Figure 3:**
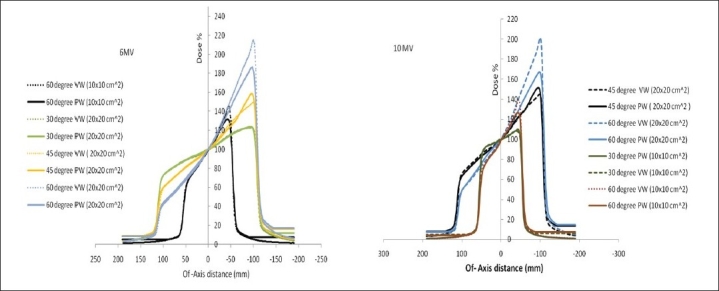
Comparison of wedge dose profiles at a depth of 10 cm for 30°, 45° and 60° wedge angles and 10×10 cm2 and 20×20 cm2 field sizes

Figure 3 shows off-axis profiles in the wedge direction at 10-cm depth for the 30°, 45° and 60° wedges with field sizes of 10 × 10 cm^2^ and 20 × 20 cm^2^ for 6- and 10-MV beams. Off-axis profiles in the wedge direction for 10 × 10 cm^2^ and 20 × 20 cm^2^ show the effect of field size on the peripheral dose (toe region). The VW off-axis profiles match well with those of the PW, except at the toe region for large wedge angles with field sizes of 10 × 10 cm^2^ and 20 × 20 cm^2^ . As can be seen in Figure 3, PW field has significantly higher doses outside the field than VW and open fields. For the field size of 10 × 10 cm^2^, peripheral doses for VW fields are less than those for the open field. For the field size of 20 × 20 cm^2^, peripheral doses on the heel side of the VW fields are, in general, smaller than those for the open field, and peripheral doses on the toe side are greater than those for the open field. The difference in peripheral doses between VW and PW increases from 5% to 8% at the toe side for field size of 10 × 10 cm^2^ in the 10-MV photon beam for wedge angles 30°, 45° and 60°, respectively.

## Discussion

### Wedge factors: Field size dependence

Figure 1 shows that normalized PWF and VWF increase slightly with the field size. Within the experimental uncertainty, our data are consistent with literature, which has shown an increase of PWF[5,11–13] and VWF[3,25,27] with field size. In general, field size dependence of PWF is attributed to the introduction of scattered-photon flounce by the wedge.[12] The number of scattered photons increases with the irradiated wedge volume, which increases with the field size. Photons scattered from a physical wedge cannot explain an increase in a VWF with field size. Gibbons and Vassy[27] have recently used a model, originally developed for EDW,[22] to predict VWFs for symmetric and asymmetric fields. This model accounts for the dose contribution to the calculation point due to additional monitor units (MUs) on the “toe” side of the wedge field. It is pointed out that the number of additional MUs significantly increases with increasing field size. Therefore, VWF increases with increasing field size, particularly for large wedge angles. The model also predicts a quadric dependence of VWF on field size, particularly for larger attenuation coefficients (*cμ*), that is, low-energy beams and larger wedge angles. Alternatively, van Santvoort[4] has suggested that the increase of VWF with field size and wedge angle is probably due to transmission through the moving collimator jaw and extrafocal radiation under this jaw. For large field sizes and wedge angles, the effect of transmission and extrafocal radiation would be larger since a relatively larger portion of the MUs is given while the jaw is moving. Differences in MUs and treatment delivery times between PW and VW are not as large as those of absolute values of WF, because the difference in the absolute values of WF between VW and PW is partially due to the fact that the MUs for VW fields are redefined. Consider using the traditional definition of MU for VW [that is, use the total MUs delivered by the linear accelerator, MU _toe_, not the central axis MUs, MU(0), to define the VWF. The effective VWF is then proportional to the measured VWF scaled by MU(0)/ MU _toe_.

(2) $${\mathrm{VWF}}_{\mathrm{eff}}\;\left(\alpha ,d,s,E\right)\;=\;{\mathrm{VWF}}_{\mathrm{meas}}\;\times \;\mathrm{MU}\left(0\right)/{\mathrm{MU}}_{\mathrm{toe}}\;=\;{\mathrm{VWF}}_{\mathrm{meas}}\;\times \;\exp\left(-\mathrm{cµ\gamma}\;\mathrm{static}\;\tan\;\alpha \right)$$

The effective VWF defined in equation (2) mimics the traditional definition of the physical WF (PWF). It should be pointed out that the second term in the above equation is the effective VWF when the “MU fraction” approximation[22] is used. The effective VWF decreases nearly exponentially with the field size because the total MUs increase exponentially with the field size for a given VW angle. EDW factors have been reported to have similar field size dependence.[20,24] On the other hand, the beam-on time required to deliver large VW angles with large field sizes may be more than that for PW, since VW uses variable dose rates.[3]

### PDD and depth dependence of WF

Differences in PDD between open and wedge fields, or depth dependence of WF, have been observed for both PWF and VWF, particularly for the 6-MV beam with a large wedge angle and large field sizes. For VW, similar results were reported by Desobry *et al*.,[26] who developed a model of energy fluence imbalance across the wedge direction, as mentioned in the previous section, to explain the dependence of VWF on depth. Relative to an open field with constant unit fluence, the wedge fluence is deficient on the heel side and excessive on the toe side. The amount of excessive fluence is always more than the amount of deficient fluence for the exponentially shaped fluence curve as described by eq. (1). Moreover, the difference between the excessive and the deficient fluences increases with field size and wedge angle. Since the majority of the excessive energy fluences was laterally located, its dose contribution to the central axis occurs almost entirely at depth.[26] Similar differences in PDD between DW/ EDW and open fields were also observed.[14,15,19,24] It was considered as clinically insignificant.[16] Van Santvoort[3] suggested that there was no depth dependence for VWFs since their measured VW PDDs were almost identical to open field PDDs and argued that it was consistent with the absence of a wedge filter that hardened the beam. For PW, it is generally accepted that the depth dependence of WF is due to beam hardening, especially for low-energy megavolt photon beams such as 6 MV.[11,12] In contrast, Kalend *et al*.[8] suggested that nearly half of the increase in the WF at depth came from the phantom scatter due to dose gradient induced by the wedge filter in the medium for Co-60, 4- and 8-MV beams. Bar-Deroma and Bjarngard,[4] however, argued that the dose-gradient scatter effect was minimal. Since there is no beam hardening of the VW fields, our data qualitatively supports the explanations of Desobry *et al*.[26] for VW and contributions of phantom scatter. Regardless of the physical explanation, we agree with Klein *et al*.,[15] that these changes in depth dose with a VW field are clinically insignificant.

### Dose profiles

We have carefully compared VW dose profiles with those of PW because we have extensive clinical dosimetric experiences with PW. The comparison would provide intuitive insights into dose distributions of VW for medical physicists and dosimetrists familiar with the dose distributions of PW. In general, dose profiles in the wedge direction of VW fields match well with those of PW fields, as shown in Figure 3. Differences in wedge profiles are observed for fields with large wedge angles and field sizes. For the 45° wedge, it is found that the PW has a higher peak in the toe area than VW, especially for field size 20 × 20 cm^2^ . The other notable difference is for the 60° wedge in the 6- and 10-MV beams with the field size of 20 × 20 cm^2^, where the VW rises slightly higher at the last 0.7 cm of the toe. For the 60° wedge in the 6- and 10-MV beams, the difference in dose profiles between VW and PW is probably clinically insignificant, considering the fact that it is only at the last 0.7 cm of the toe. For the 45° PW, the larger peak is probably due to the fact that it is designed to have the largest field width of 25 cm in the wedge direction. Siemens has recommended, “Use the normal initial gap of 1 cm for VW fields.” This would limit the largest symmetric VW field size to 21 cm. For any larger symmetric field size, the initial gap will be larger than 1 cm. For the field size of 25 cm in the VW direction, the initial gap is 3 cm. The potential of a large gap creating an excessive high dose “plateau” in the wedge toe region is a major concern for VW using large symmetric field sizes. Our results demonstrate that this concern is not justified for a wedge angle up to 45° and field sizes up to 25 cm. It is clearly illustrated by Zhu *et al*.,[31] that the initial gap of 3 cm does not create a significant hot spot. In fact, the peak at the toe of the VW is still smaller than that of the PW. Similar results have been observed for 15° and 30° wedges. Therefore, it is dosimetrically acceptable for VW fields with wedge angles up to 45° to have the initial gap of 3 cm when compared to PW fields. For 60° VW, no comparison to PW is made since the largest field size in the wedge direction for 60° PW is limited to 20 cm. The possibility of using a gap larger than 3 cm should be evaluated case by case with a treatment planning system that is capable of accurately modeling the VW dose distribution. Alternatively, for larger fields, asymmetric fields can be used for VW with a field size up to 30 cm in the wedge direction while the initial gap of 1 cm is maintained.

### Peripheral doses

Figure 3 showed that peripheral doses for PW were higher than those for VW, especially for large wedge angles. This is due to scattered radiation in the wedge filter contributing to doses outside the field. Similar results have been reported for DW,[16,17] EDW[24] and universal wedge.[31] McFarland[32] pointed out that reduction in peripheral doses would be advantageous in reducing doses to contralateral breast in the treatment of breast tangential fields.

## Conclusions

We have presented a comprehensive and direct comparison of dosimetric characteristics of Siemens VW and PW. While there is a great difference in absolute values of wedge factors, VW factors (VWFs) and PW factors (PWFs) have a similar trend as a function of field size. Dose profiles in the wedge direction match very well between VW and PW except in the toe area of large wedge angles and large field sizes. VW fields have surface doses similar to those of the open fields, while PW fields have slightly lower surface doses. Surface doses for both VW and PW increase with field size and slightly with wedge angle. VW fields in general use less monitor units than PW fields, although beam-on time may be larger for large wedge angles with large field sizes due to a variable dose rate being used for VW fields.
